# Conversion to LCP Tacrolimus Mitigates Calcineurin‐Induced Nephrotoxicity in Patients After Liver Transplantation

**DOI:** 10.1111/ctr.70602

**Published:** 2026-06-23

**Authors:** Maximilian Joseph Brol, Isabella Munske, Iyad Kabar, Philipp Stephan Althaus, Wenyi Gu, Frank Erhard Uschner, Michael Praktiknjo, Martin Sebastian McCoy, Kai‐Henrik Peiffer, Florian Rennebaum, Philipp Houben, Andreas Pascher, Hartmut H Schmidt, Jonel Trebicka, Tina Schomacher, Anna Hüsing‐Kabar

**Affiliations:** ^1^ Department of Internal Medicine B University Hospital Münster Münster Germany; ^2^ Department of General, Visceral and Transplant Surgery University Hospital Münster Münster Germany; ^3^ Department of Hepatology, Gastroenterology and Transplantation Medicine University Hospital Essen Essen Germany; ^4^ European Foundation for the Study of Chronic Liver Failure‐EF Clif Barcelona Spain

**Keywords:** CD‐ratio, kidney function, LCPT, liver transplantation, nephrotoxicitytacrolimus, renal function

## Abstract

**Background:**

Calcineurin inhibitor (CNI)‐induced nephrotoxicity after liver transplantation (LT) is linked to increased morbidity and mortality. LCPT offers a particular extended‐release formulation, potentially improving the concentration‐dose (C/D) ratio and renal outcomes. This study investigated the impact of switching from standard‐release Tacrolimus (SR‐Tac) to LCPT on C/D ratio and renal function.

**Methods:**

170 adult LT recipients (August 2008–March 2020) treated with tacrolimus for at least two years were included. In this single‐center, retrospective analysis, 63 patients converted to LCPT, while 107 patients continued on SR‐Tac. Clinical data were collected every three months over 24 months.

**Results:**

At baseline, median C/D ratios were similar between groups (*p* = 0.553), the LCPT group showed significantly higher C/D ratios compared to SR‐Tac during both the first (*p* = 0.003) and second year (*p* = 0.004). While LCPT‐treated patients showed an increased mean estimated glomerular filtration rate (eGFR), the SR‐Tac group exhibited a progressive decline at each follow‐up (mean decline: −5.4 mL/min/1.73m^2^ at 24 months, *p*<0.001). Logistic regression identified switch to LCPT (*p*<0.001), female sex (*p* = 0.043), and baseline eGFR (*p* = 0.038) as significant predictors of eGFR change.

**Conclusion:**

Conversion to LCPT may improve renal function in LT recipients over two years, suggesting potential long‐term nephroprotection without compromising graft function.

**Abbreviations**
ALT: Alanine aminotransferase
AST: Aspartate aminotransferase
BMI: Body mass index
C/D: Concentration‐to‐dose ratio
C_0_
: Trough tacrolimus concentration
CKD: Chronic kidney disease
CNI: Calcineurin inhibitor
eGFR: Estimated glomerular filtration rate
ER: Extended‐release
INR: International normalized ratio
IPTW: Inverse Probability of Treatment Weighting
IR: Immediate‐release
KDIGO: Kidney Disease: Improving Global Outcomes
LCPT: Life‐Cycle Pharma tacrolimus
LT: Liver transplantation
MASH: Metabolic dysfunction‐associated steatohepatitis
MMF: Mycophenolate mofetil
mTOR: Mammalian target of rapamycin
SD: Standard deviation
SEM: Standard error of the mean
SR‐Tac: Standard‐release tacrolimus
Tac: Tacrolimus
TBIL: Total bilirubin
ΔeGFR: Change in estimated glomerular filtration rate

## 1 Introduction

Hospitalized patients after liver transplantation (LT) with acute kidney injury showed the highest in‐hospital mortality compared to all other complications after LT [1]. Calcineurin inhibitors (CNIs), particularly tacrolimus, are crucial to immunosuppressive regimens after LT, significantly reducing the risk of acute and chronic rejection [2]. Despite their efficacy, long‐term use of CNIs is frequently complicated by nephrotoxicity, which has emerged as a leading cause of chronic kidney disease (CKD) in liver transplant recipients [3]. CNI‐induced nephrotoxicity may present acutely through reversible vasoconstriction of renal arterioles or chronically via irreversible structural damage, including interstitial fibrosis, tubular atrophy, and global glomerular sclerosis [4]. Consequently, kidney function preservation has become a critical concern in the long‐term management of liver transplant patients.

Tacrolimus is available in immediate‐release (IR) and extended‐release (ER) formulations. While both formulations are pharmacologically equivalent in terms of efficacy, they differ in pharmacokinetics. ER‐tacrolimus provides more stable blood concentrations with a lower peak‐to‐trough fluctuation, potentially reducing CNI‐associated toxicity and improving patient adherence [5, 6]. These properties have led to the hypothesis that ER formulations may be less nephrotoxic, especially in patients with known risk factors for CNI‐induced renal impairment. LCP tacrolimus (LCPT) is a novel tacrolimus formulation using MeltDose technology which reduces the drug's particle size from approximately 10 µm to submicron levels (< 0.1 µm) [7]. Data on long‐term outcomes of LCPT on renal function in patients after LT remain scarce.

An important pharmacokinetic marker used in assessing tacrolimus exposure and its impact on renal function is the concentration‐to‐dose ratio (C/D ratio)—defined as the trough blood concentration (ng/mL) divided by the daily dose (mg). A low C/D ratio is indicative of a fast tacrolimus metabolism, requiring higher doses to achieve therapeutic levels [8]. Fast metabolizers are thought to experience higher peak drug concentrations and greater intrarenal exposure, potentially exacerbating nephrotoxicity despite therapeutic trough levels [9]. Conversely, slow metabolizers, who maintain adequate trough levels at lower doses, may be less prone to CNI‐induced renal injury.

The variability in tacrolimus metabolism, largely influenced by genetic polymorphisms such as CYP3A5 expression, further complicates the management of immunosuppression [10]. Recognizing these metabolic phenotypes has important clinical implications, as fast metabolizers may benefit more from ER formulations that flatten the pharmacokinetic profile and minimize toxic peaks.

Given the burden of CKD in liver transplant recipients and the availability of alternative tacrolimus formulations, strategies that optimize immunosuppression while reducing nephrotoxicity are urgently needed [3, 11]. A recently published Delphi consensus highlighted the urgent need for studies investigating the effects of conversion to LCPT on renal function [12]. This study aims to investigate whether switching from standard‐release tacrolimus (SR‐Tac) to LCPT improves renal function in liver transplant recipients, with a particular focus on the C/D ratio and its role in identifying fast versus slow metabolizers.

## 2 Methods

### 2.1 Study Design and Participants

**FIGURE 1:**
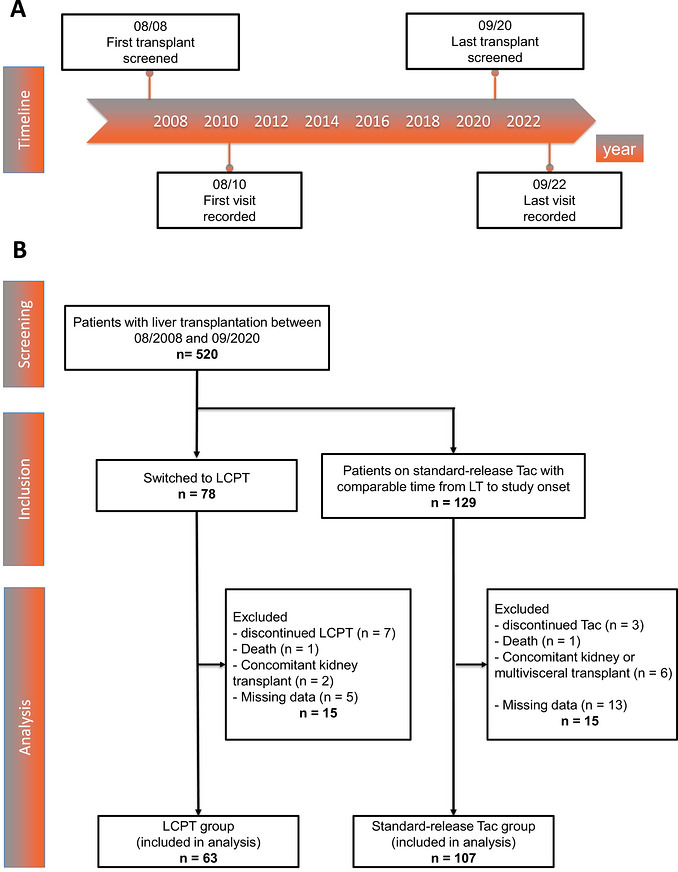
Study timeline and population flow. (A) Timeline illustrating the screening period and duration of follow‐up assessments. (B) Flow diagram of the study cohort. A total of 520 liver transplant recipients were screened of which 170 were included in the final analysis. FU, follow‐up; LCPT, life‐cycle pharma tacrolimus; LT, liver transplantation; Tac, tacrolimus.

This single‐center retrospective study was conducted in the hepatology outpatient service at the Department of Internal Medicine B at the University Hospital of Münster in Münster, Germany. Patients above the age of 18 who underwent a liver transplant were screened from August 2008 to September 2020. All grafts derived from donors after brain death. Inclusion criteria were: patients with previous LT, time from transplant to inclusion at least 4 months, stable graft function, patients on standard‐release tacrolimus (SR‐Tac) (including both immediate release (IR)‐ and extended release (ER)‐tacrolimus) as initial immunosuppressive medicine, age > 18, 24 month follow‐up visit between 08/2010 and 09/2022. First, all patients switched to LCPT were recruited. Second, patients who maintained on SR‐Tac with similar time from LT to study baseline were included. Further exclusion criteria were: missing data for 5 or more visits, no 24‐month follow‐up, death, interruption of tacrolimus use, current or prior solid organ transplantation other than LT. All of the enrolled patients received standard medical treatment according to our hospital's standard operating procedure. The flowchart of patient screening and enrolment is depicted in Figure 1. Recruited patients were finally assigned to two study groups: one group maintained on SR‐Tac, while the other group was switched to LCPT. Details on recruitment can be found in Supporting Material 1.

The immunosuppressive therapy in our department consist of intraoperative prednisolone (500 mg), followed by prednisolone (40 mg) on day 1 and 2 after transplant. Prednisolone is switched to oral administration as soon as possible and consecutively tapered within the next 90 days, unless there is an indication for continuous steroid therapy (e.g. LT in patients who previously suffered from autoimmune hepatitis). In tacrolimus monotherapy, the target tacrolimus trough level are: 6–10 ng/ml in the first month after LT, 6–8 ng/ml in the second and third month after LT. 5–8 ng/ml after 4 months of LT and 4–6 ng/ml six months after LT. Target levels may be individually lower, especially when a second immunosuppressive drug is administered (mycophenolate mofetil (MMF) or mTOR inhibitors).

Clinical and laboratory parameters were assessed at baseline. These included demographic data, etiologies, routine blood tests, liver, kidney and coagulation function parameters.

Data on tacrolimus administration was collected at every time point. All available tacrolimus through levels (C_0_) with the corresponding daily doses were collected at all visits. Visits were recorded every three months. Details on pre‐defined timepoints (visits) together with their respective window can be found in Supporting Material 1.

All research was conducted in accordance with both the Declarations of Helsinki and Istanbul. All research was approved by the local ethics committee (Ethik‐Kommission Westfalen‐Lippe, file reference: 2016‐046‐f‐S). Written consent was waived due to retrospective analysis.

### 2.2 Statistical Analysis

Data were presented as median with interquartile range or with minimum‐maximum or number with percentage or mean with standard deviation (SD) or standard error of the mean (SEM). Mann‐Whitney *U* test or Chi‐square test were used for intergroup comparison where appropriate. Paired *t*‐tests were used for intraindividual comparisons of variables. Univariable and multivariable binary logistic regression analyses were performed to identify independent predictors of renal improvement.

Inverse Probability of Treatment Weighting (IPTW) (WeightIt, survey and cobalt packages) was used to account for baseline differences between patients converted to LCPT and those maintained on SR‐Tac as previously described [13]. IPTW was performed using propensity scores derived from a logistic regression model including age, sex, diabetes, baseline eGFR, body mass index, arterial hypertension, baseline C/D ratio, time since LT, MMF use, and CKD stage. Stabilized weights were applied, and extreme weights were truncated at the first and 99th percentiles. Covariate balance before and after weighting was assessed using standardized mean differences, with values < 0.1 considered acceptable. Weighted regression models were then used to evaluate the association between LCPT conversion and renal outcomes.

*P* < 0.05 was considered statistically significant. Statistical analyses were performed with SPSS 29.0 (IBM Chicago, IL), R 4.6.0 and Prism V.10.0 (GraphPad, San Diego, CA, USA).

## 3 Results

### 3.1 General Characteristics of the Study Population

Between August 2008 and September 2020, a total of 520 liver transplant patients were screened, of whom 170 patients were included in the analysis. 63 were assigned to the LCPT group, while 107 patients constituted the group of patients who maintained on SR‐Tac within the observational period. In the baseline characteristics, the median age was 51 years with 95 (55.9%) patients being male. Median time from liver transplant to study start was 31 months in the entire cohort. The median BMI was 25.0 kg/m^2^. The majority of patients included underwent only one LT (150, 88.2%), while 20 (11.8%) underwent re‐liver transplantation (Table 1). Both groups were well balanced in terms of age at LT, age at study onset, time from LT to study onset, BMI, sex or number of previous LT (Table 1). Indication for LT was heterogeneous. Alcohol‐related liver disease, chronic viral hepatitis and metabolic dysfunction‐associated steatohepatitis (MASH) accounted for 45.9% of transplants in this population (Table 1).

**TABLE 1:** Demographic and clinical characteristics of patients.

Characteristic	Entire population (*n* = 170)	LCPT group (*n* = 63)	SR‐Tac group (*n* = 107)	*p*‐value
**Age at LT** (years)	51 (43–60)	51 (40–61)	52 (44–60)	0.738^a^
**Age at study onset** (years)	56 (45–63)	54 (43–65)	57 (47–63)	0.432^a^
**Time from LT to study onset** (months)	31 (8–79)	32 (6–83)	31 (9–76)	0.249^a^
**Height** (cm)	173 (167–180)	173 (168–178)	172 (166–181)	0.877^a^
**Weight** (kg)	76 (65–90)	79 (65–89)	75 (66–90)	0.505^a^
**BMI** (kg/m^2^)	25.0 (22.1–29.0)	26.2 (22.1–29.7)	24.7 (22.0–28.6)	0.385^a^
**Sex** (male/female)	95 (55.9%) /75 (44.1%)	33 (52.4%) / 30 (47.6%)	62 (57.9%) / 45 (42.1%)	0.480^b^
**Number of liver transplants**				0.093^b^
One	150 (88.2%)	59 (93.7%)	91 (85.0%)	
more than one	20 (11.8%)	4 (6.3%)	16 (14.9%)	
**Etiology** Alcohol‐related liver disease Chronic viral hepatitis Hereditary disorder (Wilson's disease, hemochromatosis, AAT‐deficiency) MASH Autoimmune disease other	32 (18.8%) 34 (20.0%) 16 (9.4%) 12 (7.1%) 36 (21.1%) 40 (23.5%)	11 (17.5%) 12 (19.0%) 7 (11.1%) 5 (7.9%) 13 (20.6%) 15 (23.8%)	21 (19.6%) 22 (20.6%) 9 (8.4%) 7 (6.5%) 23 (21.5%) 25 (23.4%)	0.989^b^

*Note:* Demographic and clinical characterization of the study cohort. Metric data are presented as mean ± standard deviation or median (IQR). Frequencies are presented as absolute and relative values. Statistical tests used.

Abbreviations: AAT, alpha1‐antitrypsin, BMI, body mass index, LT, liver transplantation, MASH, metabolic dysfunction‐associated steatohepatitis.

^a^ Mann–Whitney *U* test.

^b^ Chi‐squared test.

At baseline, 57.1% had arterial hypertension, 29.4% had diabetes and 69.4% had recorded CKD (Table 2). At baseline, about 30% had an unimpaired renal function with an estimated glomerular filtration rate (eGFR) above 90 mL/min/1.73 m^2^. 60.6% of enrolled subjects had a G2 or G3 CKD according to the guidelines of the KDIGO (Table 2). Of note, there was no statistically significant difference in terms of CKD stages between patients switched to LCPT compared to those who maintained on SR‐Tac at baseline (Table 2). The majority of patients received MMF as concomitant immunosuppressive agent (47.6%). The majority of patients were switched to LCPT for the prevention of treatment emerged adverse events (74.6%) at the discretion of the attending hepatologist. 10 patients were switched due to nephrotoxicity and 5 patients were switched due to neurotoxicity. No differences between the LCPT group and the SR‐Tac group were observed with regard to the etiology, metabolic risk factors or concomitant immunosuppression (Table 2).

**TABLE 2:** Underlying liver disease leading to LT, comorbidities and immunosuppression at baseline.

	Entire population (*n* = 170)	LCPT group (*n* = 63)	SR‐Tac group (*n* = 107)	*p*‐value
**Arterial hypertension**	97 (57.1%)	34 (54.0%)	63 (58.9%)	0.532
**Diabetes**	50 (29.4%)	19 (30.2%)	31 (29.0%)	0.870
**Hyperlipidemia**	57 (33.5%)	17 (27.0%)	40 (37.4%)	0.165
**Chronic kidney disease (CKD)**	118 (69.4%)	44 (69.8%)	74 (69.2%)	0.926
**CKD stages** G1 (≥ 90 mL/min/1.73m^2^) G2 (60–89 mL/min/1.73m^2^) G3a (45–59 mL/min/1.73m^2^) G3b (30–44 mL/min/1.73m^2^) G4 (15–29 mL/min/1.73m^2^) G5 and/or RRT (≤ 15 mL/min/1.73m^2^)		19 (30.2%) 19 (30.2%) 12 (19.0%) 11 (17.5%) 2 (3.2%) 0 (0%)	33 (30.8%) 45 (42.1%) 20 (18.7%) 6 (5.6%) 3 (2.8%) 0 (0%)	0.131
**Concomitant immunosuppression at baseline** MMF everolimus prednisolone (sole or in combination) sirolimus none	81 (47.6%) 41 (24.1%) 17 (10.0%) 5 (2.9%) 26 (15.3%)	29 (46.0%) 16 (25.4%) 6 (9.5%) 2 (3.2%) 10 (15.9%)	52 (48.6%) 25 (23.4%) 11 (10.3%) 3 (2.8%) 16 (15.0%)	0.996
**Reasons for switching to LCPT** Prevention for TEAE nephrotoxicity neurotoxicity reduction of tac blood concentration		47 (74.6%) 10 (15.9%) 5 (7.9%) 1 (1.6%)		
**Fast metabolizer** (defined as Tac‐C/D ratio <1.05 at baseline)	38 (22.4%)	18 (28.6%)	20 (18.7%)	0.135

*Note:*Frequencies are presented as absolute and relative values; p‐values are derived from the chi‐squared test. Unless otherwise specified, data refer to baseline (t_0_).

Abbreviations: C/D‐Ratio, concentration‐dose‐ratio, LCPT, Life cycle pharma tacrolimus, MMF, mycophenolate mofetil, RRT, renal replacement therapy, TEAE, treatment emerged adverse event.

### 3.2 Switching to LCPT led to a Less Need of Tacrolimus and Respective Higher C/D‐Ratios

**FIGURE 2:**
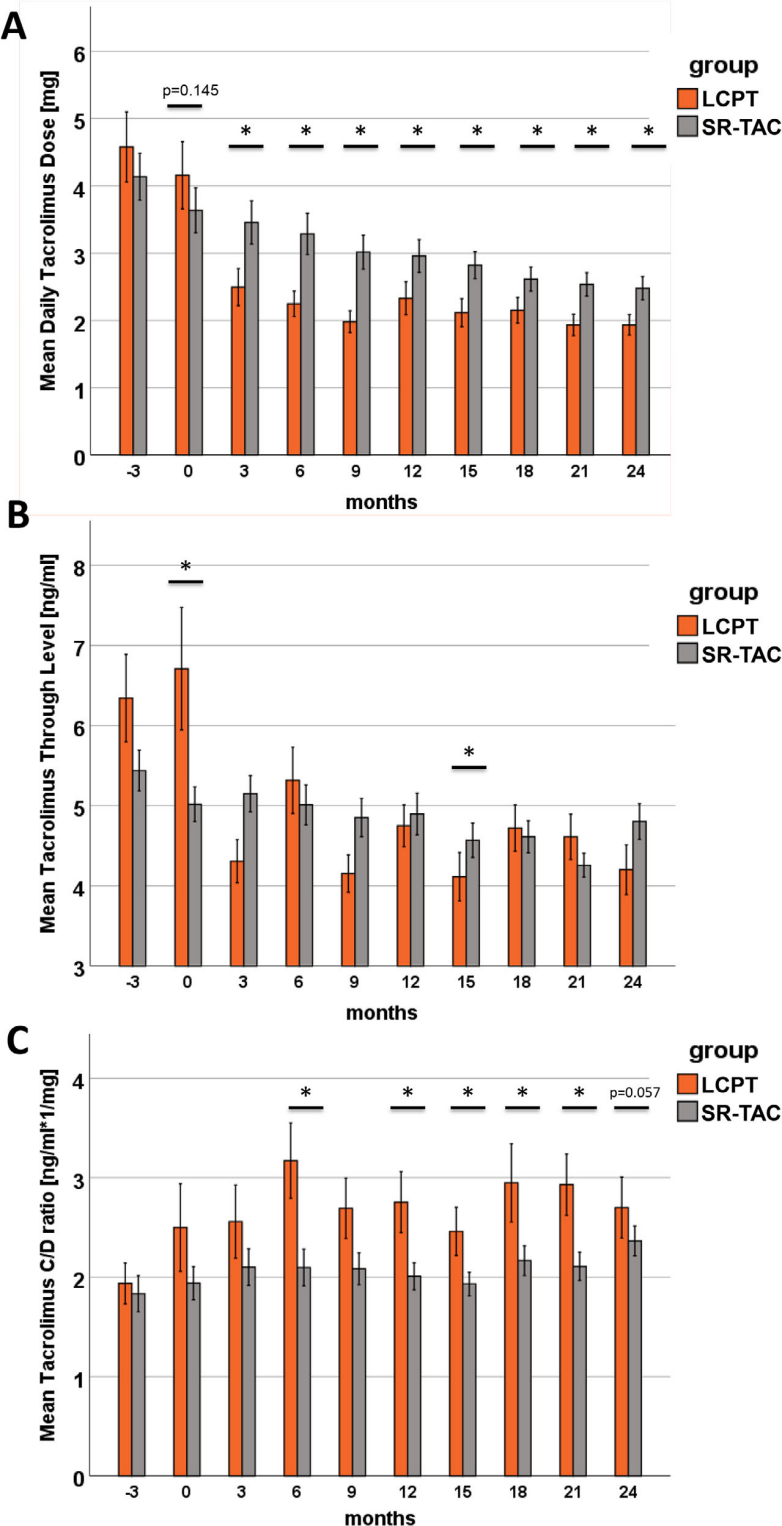
Tacrolimus dosing, trough concentrations, and concentration‐to‐dose (C/D) ratios over time. (A) Mean daily tacrolimus dose at each visit in patients switched to LCPT and those maintained on standard‐release tacrolimus (SR‐Tac). (B) Mean tacrolimus trough concentrations over time. (C) Mean tacrolimus C/D ratios across all study visits. Data are presented as mean ± standard error of the mean (SEM). Statistical comparisons between groups at each visit were performed using the Mann–Whitney U test. *p* < 0.05 was considered statistically significant. C/D, concentration‐to‐dose; LCPT, life‐cycle pharma tacrolimus; SR‐Tac, standard‐release tacrolimus.

At each follow‐up visit, the dose of tacrolimus as well as its blood through concentration were recorded. While tacrolimus doses did not differ significantly between groups at study initiation or three months prior, the LCPT group consistently received lower doses at all follow‐up visits throughout the 24‐month observation period (Figure 2A). While the median tacrolimus dose in the SR‐Tac group decreased only slightly from 3.0 mg (range: 0.5–20.0 mg) at baseline to 2.5 mg (range: 0.5–10.0 mg) after 24 months, the LCPT group demonstrated a marked reduction in median dose from 3.0 mg (range: 1.0–22.0 mg) at baseline to 1.8 mg (range: 0.8–4.0 mg) at 24 months, corresponding to a 40% dose reduction (Figure 2A, Table S1). Although tacrolimus doses were reduced following conversion to LCPT, the incidence of acute T‐cell–mediated rejection was not increased compared with the SR‐Tac group (*p* = 0.635, Table S2).

At baseline, when all participants were receiving SR‐Tac, 38 patients were classified as fast metabolizers according to their tacrolimus C/D‐ratio (Table 2). Considering individual median C/D‐ratio throughout the full observation period, only 13 patients remained fast metabolizers (Figure S1A). Interestingly, 10 fast metabolizers were in the SR‐Tac group, while only 3 were in the LCPT group (Figure S1B + SC).

Tacrolimus trough levels in the LCPT group were significantly higher at baseline when both groups received SR‐Tac but remained comparable throughout the study, except at month 15, where levels were lower in the LCPT group (3.7 vs. 4.4 ng/ml, *p* = 0.025) (Figure 2B). While baseline C/D ratios were similar, patients switched to LCPT showed higher C/D ratios from month 3 onwards, indicating more favorable exposure (Figure 2C). In five of eight follow‐ups, C/D ratios were significantly higher in the LCPT group, with a clear trend at month 24 (*p* = 0.057, Figure 2C, Table S3).

### 3.3 Switching to LCPT did not Lead to an Impairment in Liver Graft Function

For every visit, total bilirubin, transaminases (ALT and AST) as well as INR were recorded for liver function assessment. Median bilirubin was significantly higher in the SR‐Tac group compared to the LCPT group at baseline and at the 3, 6, 9 and 15, 21 and 24 months follow‐up visits (Table S4). However, all median bilirubin values were all ≤ 0.6 mg/dl, and consecutively within the reference range. Median ALT values remained in both groups within the reference range for all visits. No significant differences were observed (Table S4). Similarly, median AST values remained in both groups within the reference range for all visits. However, for four visits (baseline, 3‐, 9‐ and 15‐months follow‐up), the LCPT group had statistically significant lower levels (Table S4). Finally, INR was evaluated as a surrogate marker of hepatic synthetic function. Median INR was 1.0 for all visits and for both groups. In summary, switching from SR‐Tac to LCPT did not lead to impairment of liver graft function.

### 3.4 Switching to LCPT led to an Improvement of Renal Function

**FIGURE 3:**
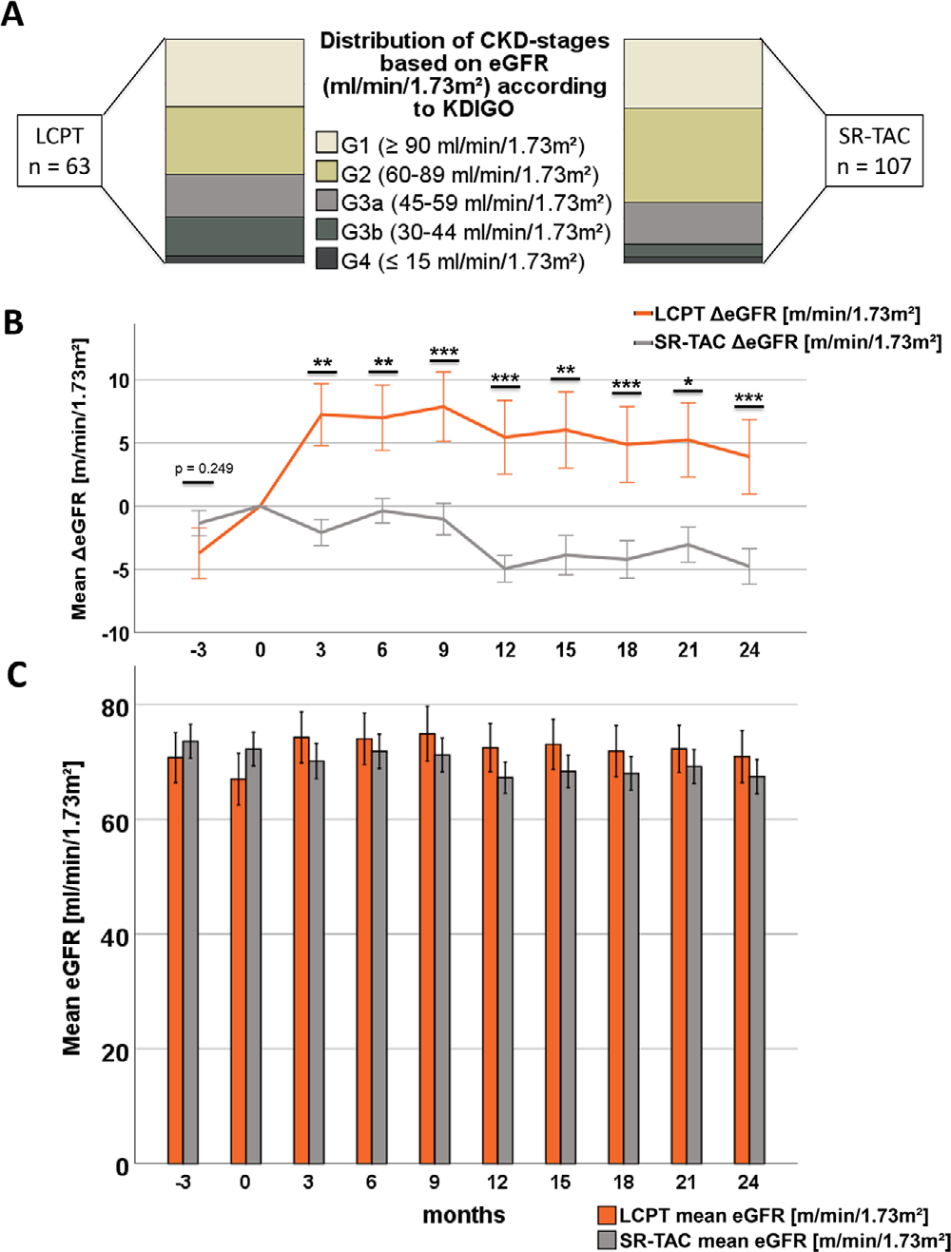
Renal function in patients treated with LCPT versus SR‐Tac. (A) Distribution of chronic kidney disease (CKD) stages at baseline based on estimated glomerular filtration rate (eGFR) according to KDIGO classification. (B) Change in eGFR (ΔeGFR slope) from 3 months before baseline to 24 months after baseline. (C) Mean eGFR values at each study visit in LCPT and SR‐Tac groups. Data in (B) and (C) are presented as mean ± SEM. P values were calculated using the Mann–Whitney U test for each timepoint (*p* < 0.05; *p < 0.01; **p < 0.001). *Abbreviations: C/D, concentration‐to‐dose; eGFR, estimated glomerular filtration rate; LCPT, Life‐Cycle Pharma tacrolimus; SR‐Tac, standard‐release tacrolimus; ΔeGFR, eGFR slope*.

Next, we assessed the effects of the conversion to LCPT on the renal function. The distribution of CKD stages according to KDIGO are visualized in Figure 3A.

Significant changes in kidney function can already be observed at the first follow‐up visit three months after baseline. In the LCPT group, a significant increase in mean eGFR was observed three months after study initiation compared to baseline (Table S5, *p* = 0.039). In contrast, the SR‐Tac group showed a significant decrease in mean eGFR at the same time point (Table S5, *p* = 0.004). This opposing trend between the groups persisted throughout the entire observation period. In the LCPT group, pairwise comparisons revealed a statistically significant increase in mean eGFR at months 3, 6, 9, 12, 15, and 18 compared to baseline. Although mean eGFR remained elevated at months 21 and 24, the differences were no longer statistically significant.

The progressive decline in mean eGFR observed in the SR‐Tac group over time is also displayed in Table S5. Pairwise comparisons of mean eGFR at each follow‐up time point with baseline revealed statistically significant reductions at every time point, indicating a consistent and significant decline in renal function. After 24 months, the mean decrease in eGFR in the SR‐Tac group was 5.4 mL/min/1.73 m^2^, while the mean increase in eGFR in the LCPT group was 2.9 mL/min/1.73 m^2^.

The eGFR values as well as the difference to baseline‐eGFR at the respective visits are shown in Figure 3B, while absolute means of eGFR are displayed in Figure 3C. While no significant difference in ΔeGFR was observed between the two study groups three months prior to baseline, significant differences in mean ΔeGFR were evident at every time point following the start of the study. The SR‐Tac group exhibited a progressively negative change in eGFR compared to baseline, with a mean ΔeGFR of −2.5 ± 8.9 mL/min/1.73 m^2^ at the 3 months follow‐up and −5.4 ± 11.0 mL/min/1.73 m^2^ at 24 months follow‐up. In contrast, the LCPT group consistently showed positive ΔeGFR values, indicating an overall increase in eGFR relative to baseline throughout the observation period. At 24 months, the mean ΔeGFR in the LCPT group was 2.9 ± 17.7 mL/min/1.73 m^2^. Compared to the SR‐Tac group's mean ΔeGFR of −5.4 ± 11.0 mL/min/1.73 m^2^, this difference was statistically significant (Figure 3B, *p* < 0.001).

### 3.5 Sex, Baseline eGFR and Switch to LCPT are Associated With Improvement of Renal Function

In order to identify predictors of eGFR improvement, we stratified the entire cohort in eGFR‐improvers (positive ΔeGFR after 24 months) (*n* = 68, 40%) and eGFR‐non‐improvers (zero or negative ΔeGFR after 24 months) (*n* = 102, *n* = 60%). Binary logistic regression was used in order to identify variables associated with improvement of renal function (Table 3). In univariable analysis, female sex, baseline eGFR and switch to LCPT were predictors of improvement of renal function after 24 months. All variables with *p* < 0.05 were included in multivariable analysis. Here, it was confirmed female sex, baseline eGFR and switch to LCPT were independent predictors of improvement of renal functions after 24 months. Among these, switch to LCPT showed the strongest association (OR 3.352 (95%‐CI: 1.706–6.590, *p* < 0.001) (Table 3).

**TABLE 3:** Uni‐ and multivariable binary logistic regression analysis with patients improving their renal function.

	Univariate binary logistic regression				multivariate binary logistic regression			
Parameter	*p*	Odd`s ratio	Confidence interval		*p*	Odd`s ratio	Confidence interval	
Age at study inclusion	0.909	1.001	0.978	1.025				
***Sex (female)***	***0.028***	***2.010***	***1.078***	***3.750***	***0.030***	***2.095***	***1.075***	***4.080***
Time from LT to tacrolimus switch	0.276	1.003	0.998	1.009				
BMI	0.775	1.008	0.953	1.067				
Re‐LT	0.321	0.575	0.193	1.716				
Cold ischemia time	0.250	1.001	1.000	1.002				
Warm ischemia time	0.693	1.003	0.988	1.019				
Etiology (ArLD)	0.116	2.272	0.816	6.321				
CKD at baseline	0.105	1.773	0.887	3.541				
Arterial hypertension	0.950	1.020	0.549	1.897				
Diabetes	0.731	1.125	0.575	2.199				
Hyperlipidemia	0.947	1.022	0.534	1.957				
MMF treatment	0.452	0.789	0.426	1.462				
Everolimus treatment	0.342	1.410	0.694	2.866				
Prednisolone treatment	0.677	0.801	0.281	2.278				
***eGFR at baseline***	***.010***	***0.983***	***0.971***	***0.996***	***0.015***	***0.984***	***0.970***	***0.997***
***Switch to LCPT***	***<0.001***	***3.489***	***1.816***	***6.701***	***<0.001***	***3.352***	***1.706***	***6.590***

Abbreviations: ArLD, alcohol‐related liver disease, BMI, body mass index, CKD, chronic kidney disease, LCPT, Life cycle pharma tacrolimus, LT, liver transplantation, MASH, metabolic dysfunction‐associated steatohepatitis. Italic ‐ Inclusion in multivariate analysis; Bold ‐ significant in multivariate analysis.

To further assess whether the renal benefit of LCPT conversion differed according to baseline renal function, we performed an additional analysis stratified by the presence of CKD. Although CKD was not independently associated with renal improvement in binary logistic regression, longitudinal analysis revealed that patients with CKD maintained on SR‐Tac experienced the most pronounced decline in eGFR, with significantly greater reductions compared with non‐CKD patients from month 9 to month 21 (Table S6). In contrast, this progressive deterioration was not observed among patients converted to LCPT, in whom eGFR remained stable in non‐CKD patients and showed an improvement in CKD patients. These findings suggest that, while CKD did not predict renal improvement per se, patients with pre‐existing CKD who remained on SR‐Tac were at the highest risk for further decrease of renal function, whereas LCPT conversion may help attenuate this decline.

In the IPTW‐adjusted analysis, conversion to LCPT was associated with significantly higher eGFR at 24 months compared with continued SR‐Tac treatment, even after adjustment for baseline eGFR (*β* = 5.82 mL/min/1.73 m^2^; 95% CI, 1.12–10.52; *p* = 0.016) (Table S7). Consistently, analysis of the change in renal function from baseline to 24 months demonstrated a significantly more favorable eGFR trajectory in the LCPT group (*β* = 5.89 mL/min/1.73 m^2^; 95% CI, 0.80–10.97; *p* = 0.024) (Table S7). Patients maintained on SR‐Tac showed an average adjusted eGFR decline of approximately 5.0 mL/min/1.73 m^2^ over 24 months, whereas renal function remained comparatively stable after conversion to LCPT. Baseline characteristics before and after IPTW as well as covariate balance before and after IPTW are displayed in Table S8 and Figure S2.

### 3.6 Patients With Diabetes Appear to Benefit Particularly From Switching to LCPT

Finally, we aimed to evaluate the potential benefits of switching to the LCPT in patients with diabetes, as this population is particularly susceptible to progressive kidney function decline after LT.

At baseline, significant differences in mean eGFR were observed between patients with diabetes and patients without diabetes within both study arms (Figure S3A: SR‐Tac group: *p* < 0.001, Figure S3B: LCPT: *p* = 0.028). In the SR‐Tac group, this significant difference in mean eGFR between patients with and without diabetes persisted at all subsequent time points (Figure S3A).

A decline in mean eGFR was observed in both subgroups within the SR‐Tac group (patients with diabetes: from 62.7 ± 19.4 mL/min/1.73m^2^ at baseline to 56.3 ± 21.4 mL/min/1.73m^2^ at 24 months; patients without diabetes: from 80.8 ± 23.2 mL/min/1.73m^2^ at baseline to 75.8 ± 22.9 mL/min/1.73m^2^ at 24 months, Figure S4, Table S9).

In contrast, subjects in the LCPT arm showed a reversed trend. While a significant difference in mean eGFR between patients with and without diabetes was present at baseline (*p* = 0.028), no significant differences were detected at subsequent time points. Irrespective of diabetes status, patients in the LCPT group exhibited a slight increase in eGFR over time, with patients with diabetes showing a more pronounced improvement (from 59.0 ± 32.7 mL/min/1.73m^2^ at baseline to 65.9 ± 30.3 mL/min/1.73m^2^ at 24 months, Table S9). Comparing mean eGFRs at baseline and at 24 months between patients with and without diabetes revealed no significant differences in both arms (Figure S4). Paired comparisons of eGFR between baseline and 24 months showed no significant differences in the LCPT group, whereas there was a statistically significant eGFR decrease for both patients with diabetes (*p* = 0.0063) and patients without diabetes (*p* = 0.0005) after 24 months (Figure S4)

In summary, these findings suggest that patients with diabetes may particularly benefit from conversion to LCPT therapy.

## 4 DISCUSSION

This study demonstrated that conversion from standard‐release tacrolimus (SR‐Tac) to LCP tacrolimus (LCPT) in liver transplant (LT) recipients leads to improved renal outcomes over a 24‐month follow‐up period. Conversion to LCPT was strongly and independently associated with improvement of eGFR after 2 years even in IPTW‐adjusted analyses. Moreover, we could show that decline of eGFR in patients with diabetes after LT and treated with tacrolimus might be mitigated after switching to LCPT.

These findings are significant with regard to the ongoing challenge of managing CNI‐induced nephrotoxicity in transplant recipients [14]. Kidney dysfunction is a common and serious complication after LT, with CKD affecting up to 30% of patients within five years post‐transplant [11]. Risk factors include pre‐existing renal impairment, perioperative acute kidney injury, early allograft dysfunction diabetes mellitus, hypertension, and long‐term CNI exposure [15, 16, 17].

A key element in understanding this benefit is the tacrolimus C/D‐ratio, a marker of individual tacrolimus metabolism and exposure, which has emerged as a predictor of renal function outcomes [8, 9]. Our findings highlight that conversion to LCPT may mitigate one of the major modifiable risk factors: tacrolimus pharmacokinetics. The more consistent drug exposure provided by LCPT likely contributes to the observed renal benefits, particularly in patients with diabetes. These data align with previous reports from kidney‐ and heart transplant patients and reinforce the potential of C/D ratio‐guided therapy in reducing renal risk [9, 18, 19].

Particularly, in kidney transplant recipients, the C/D ratio has been extensively studied. Thölking et al. first introduced the C/D ratio as a tool to stratify fast and slow metabolizers and demonstrated that fast metabolism is associated with inferior renal function despite adequate trough levels [20]. This concept was further substantiated in a recent prospective multicenter cohort study in which 101 adult kidney transplant recipients treated with LCPT were followed for 12 months [21]. The study showed that the C/D ratio in patients on LCPT was significantly higher than in historical IR‐Tac cohorts (mean 2.12 vs. 1.29; *p* < 0.001), indicating slower and more stable drug metabolism. Importantly, slow metabolizers experienced significantly better renal outcomes, with a median eGFR improvement of +7.9 mL/min at 6 months compared to −3.6 mL/min in fast metabolizers (*p* = 0.005). A Bayesian mixed‐effects model revealed that a one‐unit increase in log‐transformed C/D ratio predicted an approximate 4.5 mL/min increase in eGFR at 12 months [21].

In liver transplant recipients, evidence is growing that the C/D ratio is similarly relevant [22]. However, its application is complicated by the dual contribution of donor and recipient CYP3A4 and CYP3A5 enzyme systems in tacrolimus metabolism [23]. Nonetheless, emerging data support that fast metabolizers after LT are at greater risk for nephrotoxicity [24]. Our study confirms these findings, showing that patients who were switched to LCPT had significantly higher C/D ratios, which offers a flatter pharmacokinetic profile and reduces toxic peak exposures.

In LT patients, real‐world and clinical trial data have begun to highlight the advantages of LCPT, particularly regarding renal outcomes. Altieri et al. reported on 44 stable LT recipients converted from prolonged‐release tacrolimus to LCPT [25]. The study demonstrated that LCPT maintained stable tacrolimus trough levels with significantly lower doses and therefore improved pharmacoeconomics. Although the primary focus was safety and cost‐effectiveness, the findings implied better tolerability, which may be associated with reduced nephrotoxicity. Our study expands on this evidence by demonstrating a clear renal benefit over a 24‐month period. These findings highlight the importance of early identification and individualized management of tacrolimus metabolism profiles in LT recipients.

The presence of diabetes is of great importance since it is associated with inferior kidney function post‐transplant and an important driver to end‐stage kidney disease [26]. Notably, subgroup analysis revealed that patients with diabetes who were converted to LCPT maintained stable eGFR values over the 2‐year follow‐up, in contrast to those who remained on SR‐Tac, who exhibited a progressive decline in renal function and a statistically significant difference compared to non‐diabetic patients. This between‐group difference was no longer observed following conversion to LCPT, although it was present at baseline when all patients were still receiving SR‐Tac.

The present findings have important clinical implications. First, the C/D ratio should be considered in routine post‐transplant management to identify fast metabolizers who may benefit from early conversion to LCPT. Second, the choice of tacrolimus formulation should consider individual metabolism to balance immunosuppressive efficacy with toxicity risk. Lastly, the long‐term renal preservation afforded by LCPT may translate into improved patient survival, as already hypothesized by large register studies [26, 27].

Despite the strengths of our study, including a well‐defined cohort and long‐term follow‐up, several limitations must be acknowledged. The retrospective design precludes causal inference, and prospective randomized trials are needed to confirm our findings. Additionally, pharmacogenetic profiling (e.g., CYP3A5 genotyping) was not available, which may further enhance metabolism‐based stratification in future studies. Lastly, although conversion to LCPT was associated with improved renal function, the present study was not designed to formally evaluate cost‐effectiveness. This remains an important consideration for transplant hepatologists and healthcare systems, particularly given the long‐term nature of immunosuppressive therapy and the potential economic impact of CKD after LT.

In conclusion, conversion from SR‐Tac to LCPT in liver transplant recipients is associated with improvements in renal function, reflected by stable or improved eGFR values, particularly in patients with diabetes. These findings align with recent data in kidney transplant recipients and support the use of pharmacokinetically optimized tacrolimus formulations to reduce nephrotoxicity and improve long‐term outcomes in LT.

## Author Contributions

**Maximilian Joseph Brol:** study concept and design, acquisition of data, analysis and interpretation of data, drafting of the manuscript, critical revision of the manuscript for important intellectual content, statistical analysis, obtained funding. **Jonel Trebicka, Iyad Kabar, Tina Schomacher, Anna Hüsing‐Kabar:** study concept and design, acquisition of data, analysis and interpretation of data, critical revision of the manuscript for important intellectual content, statistical analysis, obtained funding, technical or material support and study supervision. Isabella Munske, **Wenyi Gu:** acquisition of data, analysis and interpretation of data, critical revision of the manuscript for important intellectual content, statistical analysis. **Philipp Stephan Althaus, Frank Erhard Uschner, Michael Praktiknjo, Martin Sebastian McCoy, Kai‐Henrik Peiffer, Florian Rennebaum, Philipp Houben, Andreas Pascher, Hartmut H Schmidt:** acquisition of data and critical revision of the manuscript for important intellectual content. All authors approved the final version of the manuscript.

## Conflicts of Interest

Maximilian Joseph Brol and Florian Rennebaum have received travel support and/or speaking fees from Chiesi. Jonel Trebicka has received speaking and/or consulting fees from Gore, Bayer, Alexion, MSD, Gilead, Intercept, Norgine, Grifols, Versantis, and Martin Pharmaceutical.

## Supporting information




**Supporting File1:** ctr70602‐sup‐0001‐SuppMat.docx

**Supporting File2:** ctr70602‐sup‐0002‐Material.pdf

## Data Availability

The data that support the findings of this study are available from the corresponding author upon reasonable request.
