# Supplementary material for: Conversion to LCP Tacrolimus Mitigates Calcineurin‐Induced Nephrotoxicity in Patients After Liver Transplantation
Source: Clin Transplant. 2026 Jun 23;40(6):e70602. doi: 10.1111/ctr.70602 (PMC13288323; doi:10.1111/ctr.70602)
Supplement: Supplementary file 1 — Supporting File1: ctr70602‐sup‐0001‐SuppMat.docx [file CTR-40-e70602-s002.docx]

*Supp. Table 1: Course of daily tacrolimus dose*

|  | **LCPT**  (n = 63) | **SR-Tac**  (n = 107) | **p-value** |
| --- | --- | --- | --- |
|  |  |  |  |
| **Daily tacrolimus dose [mg]** |  |  |  |
| 3 months before baseline | 3.0 (1.0 – 12.0) | 3.0 (0.5 – 19.0) | 0.695 |
| **At baseline (t0)** | **3.0 (1.0 – 22.0)** | **3.0 (0.5 – 20.0)** | **0.145** |
|  |  |  |  |
| After 3 months (t3) | 2.0 (0.5 – 8.0) | 3.0 (0.5 – 19.0) | **0.018** |
| After 6 months (t6) | 2.0 (0.4 – 7.0) | 3.0 (0.5 – 19.0) | **0.016** |
| After 9 months (t9) | 2.0 (0.8 – 6.0) | 3.0 (0.5 – 16.0) | **< 0.001** |
| After 12 months (t12) | 2.0 (0.8 – 7.8) | 2.5 (0.5 – 16.0) | **0.002** |
| After 15 months (t15) | 1.9 (0.4 – 5.5) | 2.5 (0.5 – 12.0) | **< 0.001** |
| After 18 months (t18) | 2.0 (0.8 – 5.3) | 2.0 (0.5 – 10.0) | **0.003** |
| After 21 months (t21) | 1.8 (0.8 – 5.0) | 2.0 (0.5 – 10.0) | **0.002** |
| **After 24 months (t24)** | **1.8 (0.8 – 4.0)** | **2.5 (0.5 – 10.0)** | **< 0.001** |

Course of daily tacrolimus dose of patients switched to LCPT and patients who maintained on standard release tacrolimus. Values are presented as median (minimum–maximum); p-values are derived from the Mann–Whitney U test, p-values below 0.05 were considered statistically significant (*).

*Supp. Table 2: Rejection rates*

|  | **LCPT**  (n = 63) | **SR-Tac**  (n = 107) | **p-value** |
| --- | --- | --- | --- |
|  |  |  |  |
| T-cell mediated rejections | 2 (3.2 %) | 5 (4.7 %) | 0.635 |

Number of graft rejections of patients switched to LCPT and patients who maintained on standard release tacrolimus. Values are presented as frequencies; p-values are derived from Chi-Square test.

*Supp. Table 3: Longitudinal course of C/D-ratio*

|  | LCPT  (n = 63) | SR-Tac  (n = 107) | p-value |
| --- | --- | --- | --- |
| Tac C/D-Ratio [ng/ml*1/mg]  3 months before baseline  At baseline (t0)  After 3 months (t3)  After 6 months (t6)  After 9 months (t9)  After 12 months (t12)  After 15 months (t15)  After 18 months (t18)  After 21 months (t21)  After 24 months (t24) | 1,43 (0,24 – 5,30)  **1,54 (0,30 – 13,45)**  2,00 (0,33 – 10,80)  2,43 (0,77 – 9,60)  2,00 (0,60 – 8,40)  2,49 (0,58 – 8,53)  2,28 (0,00 – 6,13)  2,50 (0,54 – 12,53)  2,30 (0,90 – 8,80)  2,35 (0,00 – 8,93) | 1,47 (0,32 – 9,20)  **1,69 (0,25 – 7,40)**  1,80 (0,29 – 7,70)  1,71 (0,24 – 7,50)  1,78 (0,00 – 7,20)  1,76 (0,44 – 6,40)  1,77 (0,00 – 5,40)  1,80 (0,00 – 7,20)  1,80 (0,00 – 6,00)  2,00 (0,28 – 5,93) | 0,724  **0,553**  0,300  **0,001***  0,217  **< 0,001***  **0,027***  **0,004***  **0,002***  0,057 |

C/D-ratios of patients switched to LCPT and patients who maintained on standard release tacrolimus. Values are presented as median (minimum–maximum); p-values are derived from the Mann–Whitney U test, p-values below 0.05 were considered statistically significant (*),C/D-Ratio (concentration-dose-ratio), SR-Tac (standard-release tacrolimus).

*Supp. Table 4. Liver function tests throughout the study*

|  |  | **LCPT** | **SR-Tac** | **p-value** |
| --- | --- | --- | --- | --- |
|  |  | (n = 63) | (n = 107) |  |
| **Timepoint** | **Parameter** |  |  |  |
|  |  |  |  |  |
| At baseline (t0) | ALT [U/l] | 19 (15-36) | 22 (17-34) | 0.141 |
|  | AST [U/l] | 22 (19 - 27) | 27 (22 – 35) | 0.003* |
|  | TBIL [mg/dl] | 0.5 (0.3 – 0.6) | 0.6 (0.4 – 0.8) | 0.011* |
| After 3 months (t3) | ALT [U/l] | 23 (16 – 34) | 25 (17 – 35) | 0.547 |
|  | AST [U/l] | 23 (20 – 29) | 28 (22 – 34) | 0.035* |
|  | TBIL [mg/dl] | 0.4 (0.3 – 0.6) | 0.6 (0.4 – 0.8) | 0.009* |
| After 6 months (t6) | ALT [U/l] | 20 (17 – 25) | 22 (17-30) | 0.485 |
|  | AST [U/l] | 23 (20 – 28) | 26 (21 – 34) | 0.058 |
|  | TBIL [mg/dl] | 0.5 (0.3 – 0.6) | 0.6 (0.4 – 0.9) | 0.013* |
| After 9 months (t9) | ALT [U/l] | 20 (15 - 33) | 22 (17 – 30) | 0.509 |
|  | AST [U/l] | 23 (19 - 30) | 27 (22 - 33) | 0.02* |
|  | TBIL [mg/dl] | 0.4 (0.3 – 0.6) | 0.5 (0.4 – 0.9) | < 0.001* |
| After 12 months (t12) | ALT [U/l] | 21 (18 – 35) | 21 (15 – 31) | 0.741 |
|  | AST [U/l] | 24 (20 - 30) | 25 (20 - 32) | 0.38 |
|  | TBIL [mg/dl] | 0.5 (0.3 – 0.7) | 0.6 (0.4 – 0.8) | 0.02 |
| After 15 months (t15) | ALT [U/l] | 21 (17 - 30) | 21 (17 - 32) | 0.807 |
|  | AST [U/l] | 22 (18 - 30) | 27 (21 – 34) | 0.007* |
|  | TBIL [mg/dl] | 0.4 (0.3 – 0.6) | 0.5 (0.4 – 0.8) | 0.016* |
| After 18 months (t18) | ALT [U/l] | 23 (16 - 33) | 21 (15 - 30) | 0.3 |
|  | AST [U/l] | 23 (19 - 30) | 27 (19 - 33) | 0.467 |
|  | TBIL [mg/dl] | 0.5 (0.4 – 0.7) | 0.6 (0.4 – 0.8) | 0.174 |
| After 21 months (t21) | ALT [U/l] | 20 (16 – 34) | 21 (16 – 29) | 0.656 |
|  | AST [U/l] | 23 (20 – 28) | 26 (21- 31) | 0.184 |
|  | TBIL [mg/dl] | 0.4 (0.3 – 0.6) | 0.6 (0.4 – 0.9) | 0.01* |
| After 24 months (t24) | ALT [U/l] | 24 (16 – 32) | 22 (16 - 31) | 0.552 |
|  | AST [U/l] | 24 (19 – 29) | 26 (21 - 34) | 0.373 |
|  | TBIL [mg/dl] | 0.4 (0.4 – 0.6) | 0.6 (0.4 – 0.9) | < 0.001* |

ALT, AST and TBIL values of patients switched to LCPT and patients who maintained on standard release tacrolimus. Values are presented as median (IQR); p-values are derived from the Mann–Whitney U test. ALT (Alanine aminotransferase), AST (Aspartate aminotransferase), SR-Tac (standard-release tacrolimus), TBIL (total bilirubin).

*Supp. Table 5. Analysis of differences in estimated filtration rates (ΔeGFR) [ml/min/1,73 m²].*

|  | **Mean ΔeGFR** | **95% confidence interval** | | **p-value** |
| --- | --- | --- | --- | --- |
|  |  | **lower** | **upper** |  |
|  |  |  |  |  |
| **LCPT** |  |  |  |  |
| -3 months vs. baseline | 2.5 | -0.1 | 5.1 | 0.06 |
| 3 months vs. baseline | 4 | 0.2 | 7.8 | **0.039** |
| 6 months vs. baseline | 4.7 | 0.6 | 8.7 | **0.024** |
| 9 months vs. baseline | 5.9 | 1.7 | 10.1 | **0.006** |
| 12 months vs. baseline | 4.7 | 0.6 | 8.8 | **0.027** |
| 15 months vs. baseline | 5.1 | 0.7 | 9.6 | **0.025** |
| 18 months vs. baseline | 4.3 | 0 | 8.5 | **0.049** |
| 21 months vs. baseline | 3.2 | -2.4 | 8.9 | 0.257 |
| 24 months vs. baseline | 2.9 | -1.6 | 7.3 | 0.205 |
|  |  |  |  |  |
| **SR-Tac** |  |  |  |  |
| -3 months vs. baseline | 0.8 | -0.8 | 2.4 | 0.328 |
| 3 months vs. baseline | -2.5 | -4.3 | -0.8 | **0.004** |
| 6 months vs. baseline | -2.5 | -2.3 | 1.1 | 0.486 |
| 9 months vs. baseline | -2.8 | -5 | -0.5 | **0.017** |
| 12 months vs. baseline | -5.3 | -7.2 | -3.5 | **< 0.001** |
| 15 months vs. baseline | -2.9 | -5.5 | -0.3 | **0.028** |
| 18 months vs. baseline | -4.3 | -6.8 | -1.8 | **0.001** |
| 21 months vs. baseline | -3.6 | -6.1 | -1.2 | **0.004** |
| 24 months vs. baseline | -5.4 | -7.5 | -3.3 | **< 0.001** |
|  |  |  |  |  |

P-values are derived from paired t-tests comparing eGFR at the respective follow-up or pre-visit (months before/after baseline) with baseline eGFR within each group. The reported mean values represent the delta between months and t₀, with positive values indicating an increase and negative values indicating a decrease in eGFR relative to baseline. All values are expressed in ml/min/1.73 m². P-values below 0.05 were considered statistically significant. SR-Tac (standard-release tacrolimus).

*Supp. Table 6. Course of differences in eGFR change in patients with and without CKD at baseline switched to LCPT vs. maintained on standard-release tacrolimus*

| Time point | LCPT | | | Standard release Tacrolimus | | |
| --- | --- | --- | --- | --- | --- | --- |
|  | **Patients with CKD**  **(n=44)** | **Patients without CKD**  **(n=19)** | **p-value** | **Patients with CKD**  **(n=74)** | **Patients without CKD**  **(n=33)** | **p-value** |
| (ΔeGFR) [ml/min/1,73m²] |  |  |  |  |  |  |
| After 3 months  After 6 months  After 9 months  After 12 months  After 15 months  After 18 months  After 21 months  After 24 months | 6 ± 17  6 ± 18  7 ± 18  7 ± 17  8 ± 19  7 ± 18  6 ± 21  4 ± 19 | 1 ± 8  2 ± 9  3 ± 10  -1 ± 13  -1 ± 8  -1 ± 10  -4 ± 14  -1 ± 13 | 0.393  0.616  0.875  0.154  0.025*  0.084  0.217  0.315 | -4 ± 10  -2 ± 9  -7 ± 12  -9 ± 10  -8 ± 12  -11 ± 14  -9 ± 14  -8 ± 14 | -2 ± 8  0 ± 8  -1 ± 11  -4 ± 9  0 ± 12  -2 ± 11  -1 ± 10  -4 ± 10 | 0.880  0.189  0.027*  0.012*  0.006*  0.004*  0.023*  0.139 |

Changes in estimated glomerular filtration rate (**Δ**eGFR; mL/min/1,73m²) in patients with diabetes and patients without diabetes in patients switched to LCPT and in patients who maintained on standard-release tacrolimus. Values are presented as mean ± standard deviation; p-values are derived from Mann Whitney U-test, p-values below 0.05 were considered statistically significant (*). CKD – chronic kidney disease.

*Supp. Table 7. Inverse probability of treatment weighting (IPTW)-adjusted analysis for association between LCPT conversion and renal function at 24 months after liver transplantation.*

| Model | Outcome | LCPT effect estimate with Standard Error (β + SE) | 95% Confidence interval | p-value | Clinical interpretation |
| --- | --- | --- | --- | --- | --- |
| IPTW-adjusted model | Change in eGFR from baseline to 24 months | **+5.89** ± 2.38 mL/min/1.73 m² | 0.80 to 10.97 | 0.024* | LCPT switch was associated with a significantly more favorable renal function trajectory over 24 months |
| IPTW-adjusted model additionally adjusted for baseline eGFR | eGFR at 24 months | **+5.82** ± 2.58 mL/min/1.73 m² | 1.12 to 10.52 | 0.016* | LCPT switch was associated with significantly higher renal function at 24 months compared with continued SR-Tac |

IPTW was applied to account for baseline differences between patients converted to LCPT and those maintained on standard-release tacrolimus. The propensity model included age, sex, diabetes mellitus, body mass index, arterial hypertension, baseline tacrolimus concentration-to-dose ratio, time since liver transplantation, mycophenolate mofetil use at baseline, and baseline CKD stage, the second further included baseline eGFR. Weighted linear regression models were used to assess the association between LCPT conversion and renal outcomes. Positive β values indicate higher eGFR or a more favorable change in eGFR in the LCPT group compared with the SR-Tac group. β (regression coefficient), CI (confidence interval), eGFR (estimated glomerular filtration rate), IPTW (inverse probability of treatment weighting), SE (standard error).

*Supp. Table 8. Baseline characteristics before and after inverse probability of treatment weighting.*

| Variable | Unweighted LCPT conversion (n = 63) | Unweighted continued SR-Tac (n = 107) | SMD | Weighted LCPT conversion (n = 62.99) | Weighted continued SR-Tac (n = 105.29) | SMD |
| --- | --- | --- | --- | --- | --- | --- |
| Age, years | 47.84 ± 16.49 | 50.07 ± 12.20 | 0.153 | 49.49 ± 15.74 | 49.55 ± 12.47 | 0.004 |
| Male sex, n (%) | 33 (52.4) | 62 (57.9) | 0.112 | 35.9 (57.1) | 59.2 (56.3) | 0.016 |
| Diabetes mellitus, n (%) | 19 (30.2) | 31 (29.0) | 0.026 | 17.2 (27.4) | 29.8 (28.3) | 0.021 |
| Baseline eGFR, mL/min/1.73 m² | 70.84 ± 28.24 | 75.54 ± 23.59 | 0.181 | 73.87 ± 27.22 | 74.29 ± 24.74 | 0.016 |
| BMI, kg/m² | 26.71 ± 6.07 | 25.75 ± 4.97 | 0.174 | 26.05 ± 5.45 | 25.77 ± 5.08 | 0.054 |
| Arterial hypertension, n (%) | 34 (54.0) | 63 (58.9) | 0.099 | 34.5 (54.8) | 59.3 (56.3) | 0.030 |
| Baseline C/D ratio | 2.19 ± 2.17 | 1.90 ± 1.14 | 0.165 | 1.99 ± 1.69 | 1.91 ± 1.16 | 0.052 |
| Time since LT, months | 48.83 ± 55.49 | 52.24 ± 54.10 | 0.062 | 46.58 ± 55.05 | 49.41 ± 52.77 | 0.052 |
| MMF use at baseline, n (%) | 29 (46.0) | 52 (48.6) | 0.051 | 29.0 (46.0) | 50.1 (47.5) | 0.031 |
| CKD stage at baseline, n (%) |  |  | 0.410 |  |  | 0.039 |
| No CKD | 19 (30.2) | 33 (30.8) |  | 18.6 (29.5) | 32.4 (30.7) |  |
| CKD stage 1 | 19 (30.2) | 45 (42.1) |  | 24.1 (38.3) | 40.5 (38.5) |  |
| CKD stage 2 | 12 (19.0) | 20 (18.7) |  | 12.3 (19.5) | 19.7 (18.7) |  |
| CKD stage 3 | 11 (17.5) | 6 (5.6) |  | 6.2 (9.9) | 9.7 (9.2) |  |
| CKD stage 4 | 2 (3.2) | 3 (2.8) |  | 1.8 (2.8) | 3.1 (2.9) |  |

Baseline characteristics of patients converted to LCPT and patients maintained on SR-Tac before and after inverse probability of treatment weighting. Continuous variables are presented as mean ± standard deviation; categorical variables are presented as n (%). Weighted values represent weighted counts and percentages. Standardized mean differences were used to assess covariate balance before and after weighting, with values <0.1 considered indicative of adequate balance. BMI (body mass index), C/D (concentration-to-dose ratio), CKD (chronic kidney disease), eGFR (estimated glomerular filtration rate), LCPT (Life-Cycle Pharma tacrolimus), LT (liver transplantation), MMF (mycophenolate mofetil), SMD (standardized mean difference), SR-Tac (standard-release tacrolimus).

*Supp. Table 9. Course of eGFR in patients with and without diabetes switched to LCPT vs. maintained on standard-release tacrolimus*

| Time point | LCPT | | | Standard release Tacrolimus | | |
| --- | --- | --- | --- | --- | --- | --- |
|  | **Patients with diabetes**  **(n=19)** | **Patients without diabetes**  **(n=44)** | **p-value** | **Patients with diabetes**  **(n=31)** | **Patients without diabetes**  **(n=76)** | **p-value** |
| (eGFR) [ml/min/1,73m²] |  |  |  |  |  |  |
| Three months before baseline | 65.4 ± 31.6 | 76.8 ± 22.7 | 0.056 | 66,2 ± 20.8 | 80,5 ± 24.1 | 0.007* |
| At baseline (t0)  After 3 months  After 6 months  After 9 months  After 12 months  After 15 months  After 18 months  After 21 months  After 24 months | 59.0 ± 32.7  68.3 ± 31.3  68.3 ± 29.7  70.0 ± 33.1  66.8 ± 29.7  69.1 ± 30.6  65.2 ± 29.6  69.2 ± 29.4  65.9 ± 30.3 | 76.0 ± 24.7  79.0 ± 25.4  78.0 ± 24.4  79.3 ± 25.4  79.3 ± 21.6  80.0 ± 23.5  78.6 ± 23.4  75.7 ± 22.3  77.1 ± 24.5 | 0.013*  0.114  0.110  0.175  0.033*  0.108  0.059  0.587  0.071 | 62.7 ± 19.4  59.7 ± 18.4  63.7 ± 17.4  60.7 ± 18.9  56.7 ± 18.5  59.1 ± 21.2  59.1 ± 22.2  57.7 ± 21.8  56.3 ± 21.4 | 80.8 ± 23.2  79.0 ± 24.5  80.2 ± 24.2  77.8 ± 24.4  74.9 ± 22.1  78.3 ± 24.0  75.0 ± 22.8  75.4 ± 22.9  75.8 ± 22.9 | < 0.001*  < 0.001*  < 0.001*  0.001*  < 0.001*  0.001*  0.003*  0.001*  < 0.001* |

Estimated glomerular filtration rate (eGFR; mL/min/1,73m²) in patients with diabetes and patients without diabetes in patients switched to LCPT and in patients who maintained on standard-release tacrolimus. Values are presented as mean ± standard deviation; p-values are derived from Mann Whitney U-test, p-values below 0.05 were considered statistically significant (*).


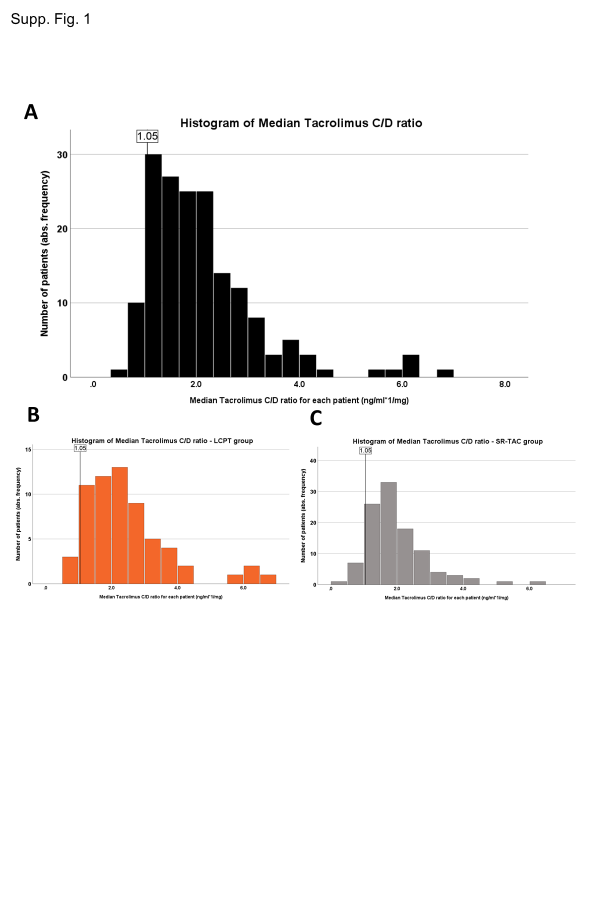
 **^**

**Supporting Figure 1. Distribution of individual median tacrolimus C/D ratios.**

(A) Histogram of individual median tacrolimus C/D ratios across all patients. A C/D ratio <1.05 was used as the threshold for identifying fast metabolizers based on existing literature.

(B) Histogram of individual median C/D ratios in the LCPT group.

(C) Histogram of individual median C/D ratios in the SR-Tac group.

*Abbreviations: C/D, concentration-to-dose; LCPT, Life-Cycle Pharma tacrolimus; SR-Tac, standard-release tacrolimus.*


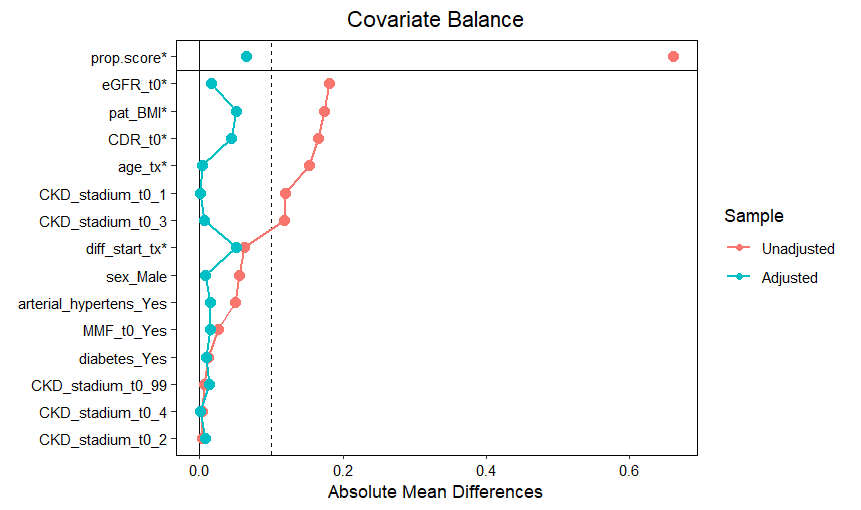


**Supporting Figure 2. Covariate balance before and after inverse probability of treatment weighting.**

Love plot showing absolute standardized mean differences for baseline covariates before and after inverse probability of treatment weighting. Unadjusted values represent covariate balance between patients converted to LCPT and those maintained on SR-Tac before weighting, whereas adjusted values represent balance after application of stabilized IPTW weights. The dashed vertical line indicates the predefined threshold of 0.1, below which covariate balance was considered acceptable. After weighting, all included baseline covariates were below this threshold, indicating adequate balance between treatment groups. CDR (concentration-to-dose ratio), CKD (chronic kidney disease), eGFR (estimated glomerular filtration rate), IPTW (inverse probability of treatment weighting), LCPT (Life-Cycle Pharma tacrolimus), MMF (mycophenolate mofetil), SR-Tac (standard-release tacrolimus).


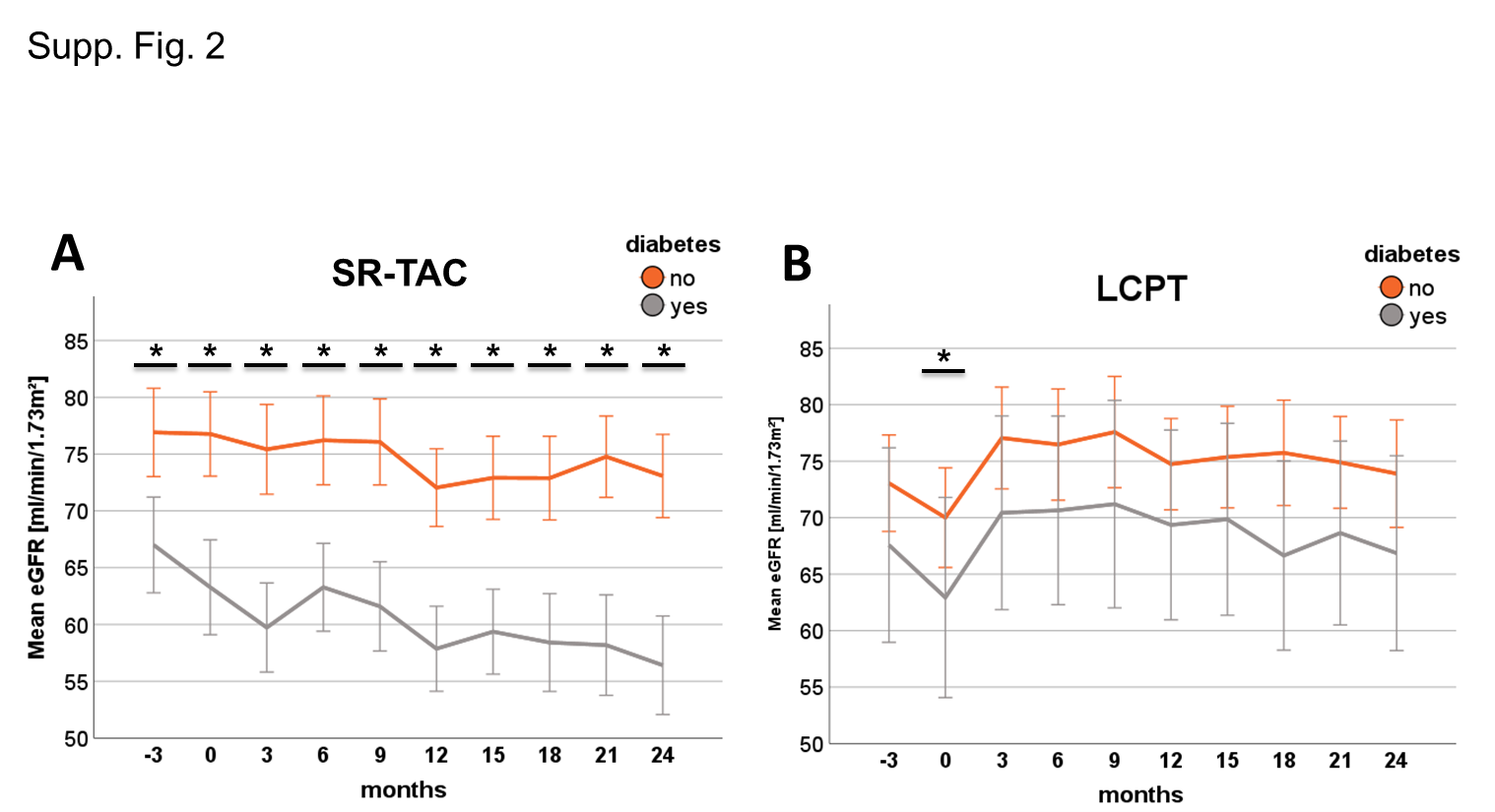


**Supporting Figure 3. Course of eGFR in patients treated with LCPT versus SR-Tac depending on presence of diabetes.**

(A) Course of estimated glomerular filtration rate (eGFR) from 3 months before baseline to 24 months after baseline in patients maintained on standard-release tacrolimus (SR-Tac) stratified by presence of diabetes.

(B) Course of estimated glomerular filtration rate (eGFR) from 3 months before baseline to 24 months after baseline in patients switched to LCPT stratified by presence of diabetes.

*Abbreviations: eGFR, estimated glomerular filtration rate; LCPT, Life-Cycle Pharma tacrolimus; SR-Tac, standard-release tacrolimus.*


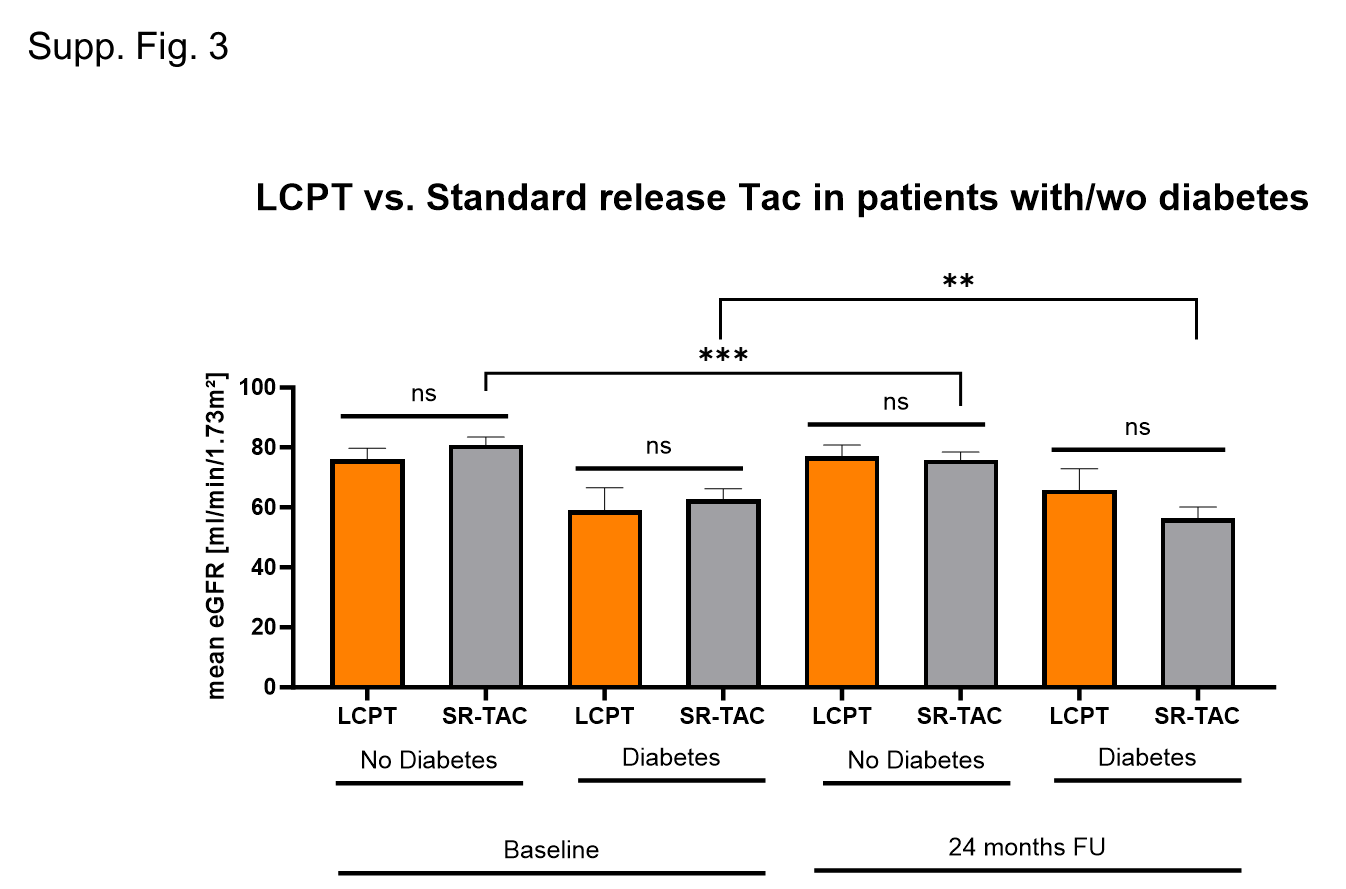


**Supporting Figure 4. eGFR at baseline and 24-month follow-up stratified by diabetes status.**

Mean eGFR at baseline and at 24 months in patients switched to LCPT and those maintained on SR-Tac, further stratified by the presence of diabetes.

Data are presented as mean ± SEM. P values were calculated using the Mann–Whitney U test. **P < 0.01; ***P < 0.001.

*Abbreviations: eGFR, estimated glomerular filtration rate; FU, follow-up; LCPT, Life-Cycle Pharma tacrolimus; SR-Tac, standard-release tacrolimus.*
