# Supplementary material for: Conversion to LCP Tacrolimus Mitigates Calcineurin‐Induced Nephrotoxicity in Patients After Liver Transplantation
Source: Clin Transplant. 2026 Jun 23;40(6):e70602. doi: 10.1111/ctr.70602 (PMC13288323; doi:10.1111/ctr.70602)
Supplement: Supplementary file 2 — Supporting File2: ctr70602‐sup‐0002‐Material.pdf [file CTR-40-e70602-s001.pdf]

**Supporting Material 1:**

## Timepoints and Data Collection

For the purpose of longitudinal analysis, standardized measurement timepoints were defined relative to a study baseline (t0). Data were collected retrospectively and assigned to specific intervals based on the date of laboratory assessments, with defined tolerance windows to accommodate variability in routine clinical sampling.

### *Definition of Timepoints*

- **t3_before**: Approximately three months prior to study baseline (t0).
- **t0 (Study Baseline)**:
    - In the **LCPT arm** (extended-release tacrolimus), t0 was defined as the date of medication switch (initiation of LCPT), corresponding to the last available laboratory values prior to the first dose of LCPT. To ensure stabilization of baseline immunosuppression, t0 was set no earlier than the fourth month after liver transplantation.
    - In the **SR-Tac arm** (standard-release tacrolimus), patients were included matched for time from LT to study baseline. t0 was aligned with the average timepoint of medication switch in the LCPT arm, to ensure comparability between study groups.
- **t3** to **t24**: Follow-up timepoints defined at 3-month intervals following t0.

### *Assignment of Laboratory Values to Timepoints*

Laboratory results were assigned to measurement timepoints according to the following tolerance windows:

- **t3_before**: 1.6 to 4.5 months before t0.
- **t0**: Laboratory values obtained on or immediately before the day of medication switch, but no earlier than 3 months post-transplantation.
- **t3**: 1.6 to 4.5 months after t0.
- **t6**: 4.6 to 7.5 months after t0.
- **t9**: 7.6 to 10.5 months after t0.
- **t12**: 10.6 to 13.5 months after t0.
- **t15**: 13.6 to 16.5 months after t0.

- **t18**: 16.6 to 19.5 months after t0.
- **t21**: 19.6 to 22.5 months after t0.
- **t24**: >22.5 to a maximum of 27 months after t0.

If individual laboratory parameters were missing at a given timepoint, values from two separate blood draws within a maximum of three weeks could be combined to complete the dataset for that timepoint. In such cases, the date of the **earlier** blood draw was used to determine the timepoint allocation.

To ensure consistency in assigning measurements at month boundaries:

- Measurements taken up to and including the 15th day of the month were considered as "x.5" months.
- Measurements from the 16th onward were assigned as "x.6" months.
